# Neuronal and astrocytic interactions modulate brain endothelial properties during metabolic stresses of in vitro cerebral ischemia

**DOI:** 10.1186/1478-811X-12-7

**Published:** 2014-01-17

**Authors:** Ganta Vijay Chaitanya, Alireza Minagar, Jonathan S Alexander

**Affiliations:** 1 Department of Molecular and Cellular Physiology, Louisiana State University Health-Shreveport, Louisiana 71103, USA; 2 Current Address: Robert M. Berne Cardiovascular Research Center, Department of Biomedical Engineering, University of Virginia, Charlottesville, VA 22908, USA; 3 Department of Neurology, Louisiana State University-Health, Shreveport, LA 71130, USA

## Abstract

Neurovascular and gliovascular interactions significantly affect endothelial phenotype. Physiologically, brain endothelium attains several of its properties by its intimate association with neurons and astrocytes. However, during cerebrovascular pathologies such as cerebral ischemia, the uncoupling of neurovascular and gliovascular units can result in several phenotypical changes in brain endothelium. The role of neurovascular and gliovascular uncoupling in modulating brain endothelial properties during cerebral ischemia is not clear. Specifically, the roles of metabolic stresses involved in cerebral ischemia, including aglycemia, hypoxia and combined aglycemia and hypoxia (oxygen glucose deprivation and re-oxygenation, OGDR) in modulating neurovascular and gliovascular interactions are not known. The complex intimate interactions in neurovascular and gliovascular units are highly difficult to recapitulate *in vitro*. However, in the present study, we used a 3D co-culture model of brain endothelium with neurons and astrocytes *in vitro* reflecting an intimate neurovascular and gliovascular interactions *in vivo*. While the cellular signaling interactions in neurovascular and gliovascular units *in vivo* are much more complex than the 3D co-culture models *in vitro*, we were still able to observe several important phenotypical changes in brain endothelial properties by metabolically stressed neurons and astrocytes including changes in barrier, lymphocyte adhesive properties, endothelial cell adhesion molecule expression and *in vitro* angiogenic potential.

## Background

Neurovascular and gliovascular units form the functional units in the brain [1-4]. These units consist of a highly intimate, organized and complex association between neurons, astrocytes, brain endothelium, pericytes and fibroblasts. Neurons are at the center of the neurovascular units and astrocytes are at the center of gliovascular units. In the gliovascular unit, astrocytes ensheath blood vessels at one end and at the other end communicate with neuronal pre- and post-synaptic processes [5,6]. While electrical and biochemical synapses play a key role in neuronal information exchange, astrocytes communicate through the gap junctions in their end foot processes regulated by extracellular and intracellular signaling [7,8]. Astrocytes also play a highly important role in neurovascular coupling [9-11]. These units work in a perfectly synchronized manner to regulate the central nervous system (CNS) biochemical and physiological processes involved in cerebral blood flow, brain energetics, blood brain barrier (BBB) properties, brain development and in responding and adapting to alterations in several systemic physiological and pathophysiological alterations [1,3,8,12-15].

At a fundamental level, according to the report of stroke progress group 2002, the neurovascular unit was defined as “*the molecular influences and cell-signaling mechanisms that characterize the interactions between circulating blood elements and the blood vessel wall, extracellular matrix, glia, and neurons (together, the neurovascular unit) during ischemic and hemorrhagic stroke*” (*Report of the Stroke Progress Research Group, 2002*; http://www.ninds.nih.gov) [3]. Understanding the interactions between these neurovascular components can define the broad spectrum of events involved in the initiation and progression of ischemia, hemorrhage, brain inflammation, brain edema, BBB dysfunction and white matter changes post stroke. This fundamental definition of neurovascular unit also described the interactions between blood vessel wall, glia and neurons and circulating blood elements, indicating the participation of peripheral immune system in the function of neurovascular unit. The brain is considered to be an immune privileged organ, where BBB functions to prevent infiltration of immune cells from the blood [16-18]. However, leukocytes were observed to be present in embryonic and adult brain in normal physiological conditions [16,19]. These neurovascular- and gliovascular-immune interactions gain more importance depending on the presence of perivascular antigen presenting cells in the inner and outer wall of the astrocyte end feet and basement membrane of endothelial cells in the perivascular space [19]. This indicates that communication with systemic immune compartment might also be a part of neurovascular and gliovascular functions [20]. However, the physiological significance for these interactions is not clear.

Occlusion of blood flow to any region of the brain results in deprivation of oxygen, glucose and nutrients to the neurovascular and gliovascular units [21-23]. This results in uncoupling of neurovascular and gliovascular units and in severe pathological alterations in the physical and physiological properties of these units. BBB breakdown results in exacerbation of vasogenic cerebral edema which is one of the most life threatening complications post stroke [24,25]. This process is greatly intensified by the production of astrocytic matrix metalloproteinases (MMPs) and by aquaporins in the astrocytic end foot process (3–5; 11; 13). Physical swelling of the astrocyte end feet process also contributes to increased BBB permeability indicating gliovascular uncoupling [7,9].One of the most important features in the neurovascular and gliovascular uncoupling during cerebral ischemia is the intercellular biochemical signaling that results in perturbations in BBB permeability [12,26,27]. Furthermore, the disturbances in the vascular wall of neurovascular and gliovascular units also trigger vascular remodeling and angiogenesis which are considered to be endogenous adaptive events post-stroke [28].

Normal brain endothelium is relatively inert to support leukocyte interactions. However, ischemia results in a phenotypical change of ‘resting’ brain endothelium to ‘reactive endothelial’ phenotype thereby supporting the infiltration of leukocytes into the infarcted brain tissue [29]. These subtle but highly orchestrated neurovascular and gliovascular-immune interactions during normal physiological conditions can therefore become reactive and chaotic due to infiltrating immune cells in the post-stroke brain due to BBB damage, increased permeability and expression of endothelial cell adhesion molecules (ECAM) that facilitates these processes [23,30,31]. Furthermore, release of damage associated molecular patterns (DAMP) from reactive astrocytes post ischemic episode may also function as a chemoattractant for leukocyte infiltration [32,33], indicating that multiple processes in neurovascular and gliovascular units during cerebral ischemia contribute to aggravation of ischemic brain damage not only from ‘inside’ but also from ‘outside’ by the participation of systemic immune responses. Increased cytokine/chemokine milieu due to reactive astrogliosis and infiltrating leukocytes greatly affect neurovascular and gliovascular responses and modulate outcome of the ischemic brain damage [21].

We have previously reported that cytokines differentially modulate brain endothelial and gliovascular barrier responses [24]. Furthermore, we have also observed that the metabolic components of cerebral ischemia (i.e. aglycemia, hypoxia and OGDR) have differential effects on brain endothelial adhesion molecule expression [25]. However, considering the intimate physical and biochemical interactions between the primary components of neurovascular and gliovascular units, it is not clear how these metabolic stresses affect neurovascular and gliovascular units. In the current study we wanted to understand how the metabolic disturbances can affect neurons and astrocytes which in turn could affect the basic characteristic properties of brain endothelium including barrier, angiogenic potential, ECAM expression and leukocyte interactions.

Since targeting a specific cell type as a therapeutic approach for stroke has received set back, therapies aimed at protecting neurovascular and gliovascular uncoupling from ischemia-reperfusion induced damage has received more attention. In order to develop efficient translational therapies, it becomes important to understand the basic pathophysiology of interactions between the components of neurovascular and gliovascular units. In the present study using 3D co-culture system with endothelial cells/neurons and endothelial cells/astrocytes representing intimate neurovascular and gliovascular coupling, we investigated the role of metabolic stresses in modulating neurovascular and gliovascular responses in affecting brain endothelial barrier, angiogenesis and leukocyte interactions via ECAM expression.

## Results

### Brain endothelial, neurovascular and gliovascular barriers are modulated by in vitro ischemic metabolic stresses

#### Brain endothelial barrier

##### Normal

**Figure 1:**
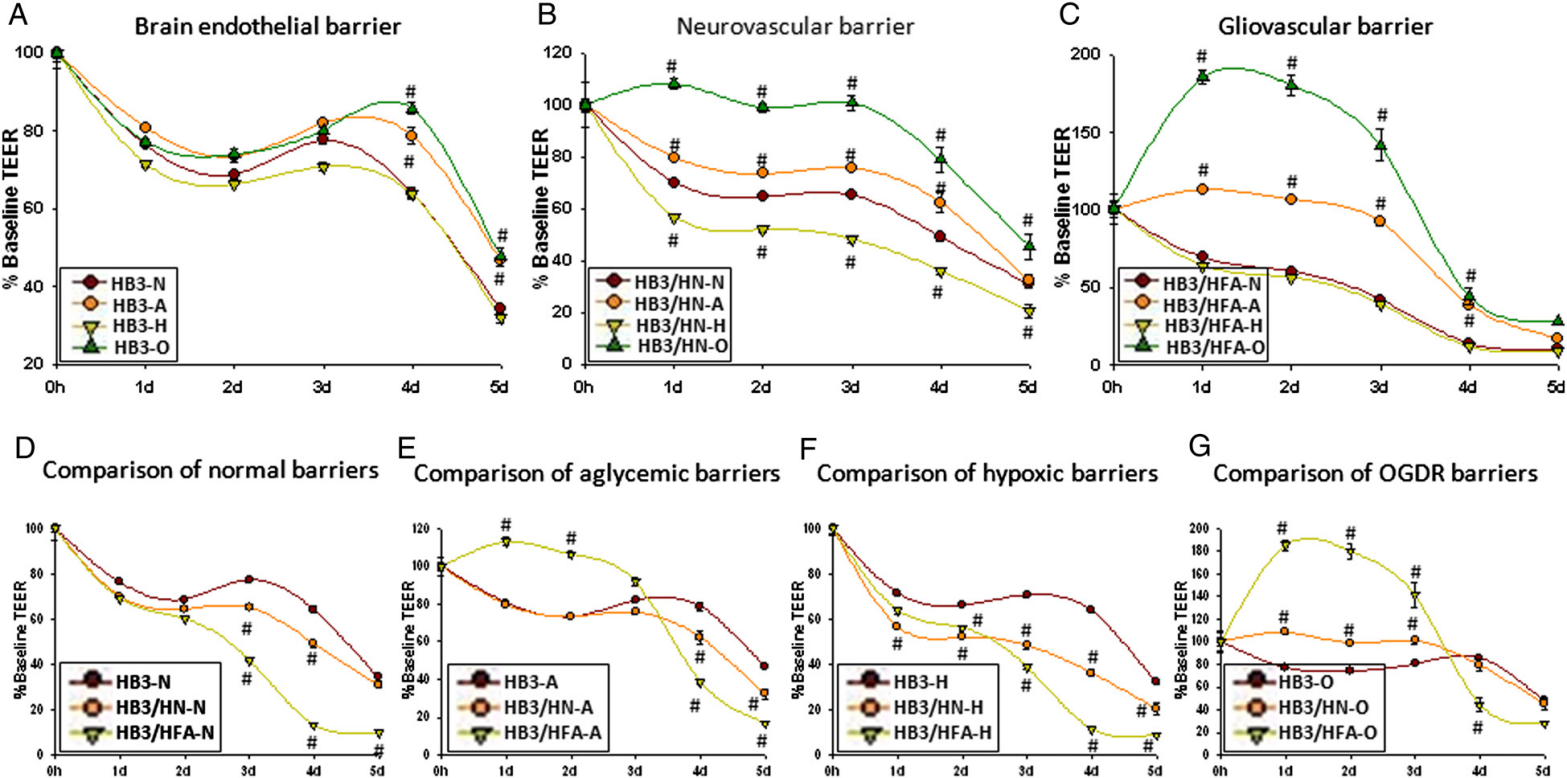
**Differential effects of metabolic stresses on brain endothelial, neurovascular and gliovascular barriers. A)***Brain endothelial barrier*. Significant differences were observed between normal and OGDR brain endothelial barrier at 4d. **B**. *Neurovascular barrier*. Significant differences between aglycemic, hypoxic and OGDR treated neurovascular barrier was observed from 1d until 5d compared to untreated neurovascular barrier. **C**. *Gliovascular barrier.* While no significant differences between untreated and hypoxic gliovascular barrier were observed, aglycemic and OGDR gliovascular barrier was significantly different from untreated from 1d to 3d. **D**. *Comparison of untreated brain endothelial, neurovascular and gliovascular barriers*. No significant differences between brain endothelial, neurovascular and gliovascular barriers were observed until 2d. However, neurovascular barrier was significantly lower compared to brain endothelial barrier until 4d and gliovascular barrier was significantly lower until 5d. **E**. *Comparison of aglycemic brain endothelial, neurovascular and gliovascular barriers*. No significant differences between aglycemic brain endothelial and neurovascular barriers were observed. However, significant increase in aglycemic gliovascular barrier was observed until 3d. Aglycemic gliovascular barrier was significantly lower at 4d and 5d compared to aglycemic brain endothelial barrier. **F**. *Comparison of Hypoxic brain endothelial, neurovascular and gliovascular barriers*. While hypoxic neurovascular barrier was significantly lower than hypoxic brain endothelial barrier from 3d, hypoxic gliovascular barrier was significantly lower from 1d until 5d. **G**. *Comparison of OGDR brain endothelial, neurovascular and gliovascular barriers*. Both OGDR neurovascular and gliovascular barriers showed a significant increase compared to OGDR brain endothelial barrier until 3d. However, at 4d OGDR gliovascular barrier was significantly lower compared to OGDR brain endothelial and neurovascular barriers. Repeated measures ANOVA from 0h baseline. Values are expressed in percent baseline at 0h ± SEM. Un-paired t-test was used to check significance between groups at the same time point. # P<0.05 is considered significantly different from respective controls at the same time point.

Under normal conditions brain endothelial barrier showed a progressive decline from 0 h baseline time point. Significant differences between normal, aglycemic and OGDR challenged brain endothelial barrier were observed at 4d and 5d. The barrier of normal brain endothelial barrier at 4d was 64.14 ± 1.4% (Figure 1A, Additional file 1: Figure S1A).

##### Aglycemia

No significant differences were observed between normal and aglycemia challenged brain endothelial barrier until 3d. However, the barrier of aglycemia challenged brain endothelial was significantly higher compared to the normal barrier at 4d and 5d. The barrier of aglycemia challenged brain endothelial at 4d was 78.74 ± 2.3% (Figure 1A, Additional file 1: Figure S1A).

##### Hypoxia

Hypoxia challenged brain endothelial barrier showed a progressive loss of barrier from 1d similar to normal brain endothelial barrier. At 4d the barrier of hypoxia challenged brain endothelial was 63.7 ± 0.9% (Figure 1A, Additional file 1: Figure S1A).

##### OOGDR

OGDR challenged brain endothelial cells showed no significant difference in the barrier compared to the normal barrier until 3d. However, OGDR brain endothelial barrier showed significant differences from 4d and 5d. At 4d the barrier of OGDR HBMEC3 was 85.83 ± 1.6% (Figure 1A, Additional file 1: Figure S1A).

The rank order of barrier in this experiment was observed to be N=H<A<O.

#### Neurovascular barrier

##### Normal

Neurovascular co-culture showed a progressive loss of barrier from 0 h baseline time point at normal conditions. Significant differences in normal and metabolic challenged HBMEC3/SHSY-5Y co-cultures were observed until 5d. At 4d the normal neurovascular barrier was 49.02 ± 1.5% (Figure 1B, Additional file 1: Figure S1B).

##### Aglycemia

The barrier of aglycemia challenged neurovascular co-culture was significantly higher compared to the normal barrier until 4d. At 4d aglycemia challenged neurovascular barrier was 62.22 ± 3.8% (Figure 1B, Additional file 1: Figure S1B).

##### Hypoxia

The barrier of hypoxia challenged neurovascular co-culture barrier was significantly lower at all-time points compared to normal or aglycemia or OGDR. At 4d hypoxia challenged neurovascular barrier was 36.13 ± 1.3% (Figure 1B, Additional file 1: Figure S1B).

##### OGDR

The barrier of OGDR challenged neurovascular co-culture barrier was significantly higher compared to all other treatment conditions. At 4d OGDR challenged neurovascular barrier was 78.89 ± 5.0% (Figure 1B, Additional file 1: Figure S1B).

The rank order of barrier in this experiment was H<N<A<O.

#### Gliovascular barrier

##### Normal

Normal gliovascular co-culture barrier showed a progressive loss of barrier from 0 h baseline time point. Significant differences between normal, aglycemic and OGDR challenged gliovascular co-culture barrier were observed until from 1d until 4d. At 4d normal gliovascular barrier was 13.26 ± 0.4% (Figure 1C, Additional file 1: Figure S1C).

##### Aglycemia

Aglycemia challenged gliovascular barrier was significantly higher compared to normal gliovascular barrier until 4d. At 4d aglycemia challenged gliovascular barrier was 38.58 ± 1.6% (Figure 1C, Additional file 1: Figure S1C).

##### Hypoxia

Hypoxia challenged gliovascular co-culture barrier showed progressive loss from 0 h time point, similar to normal gliovascular co-culture barrier. At 4d hypoxia challenged gliovascular barrier was 11.4 ± 0.2% (Figure 1C, Additional file 1: Figure S1C).

##### OGDR

OGDR challenged gliovascular co-culture showed a dramatic increase in from 0 h baseline time point until 4d. At 4d OGDR challenged gliovascular barrier was 44.05 ± 5.7% (Figure 1C, Additional file 1: Figure S1C).

The rank order of barrier in this experiment was N=H<A<O.

#### Comparison between vascular, neurovascular and gliovascular barriers

##### Normal

A progressive decline in brain endothelial, neurovascular and gliovascular barriers were observed under normal conditions. While neurovascular barrier was significantly lower than brain endothelial barrier at 3d and 4d time points, gliovascular co-cultures showed a significantly decreased barrier from 3d to 5d compared to brain endothelial barrier under normal conditions. The barrier differences among these barriers were maximal at 4d. Brain endothelial barrier at 4d was 64.14 ± 1.5%, neurovascular barrier was 49.02 ± 1.5% and gliovascular barrier was 13.26 ± 0.4% (Figure 1D, Additional file 1: Figure S1D).

The rank order in these comparisons was HBMEC3>HBMEC3/SHSY-5Y>HBMEC3/HFA.

##### Aglycemia

When challenged with aglycemic stress, the brain endothelial, neurovascular and gliovascular barriers showed differential responses in a time dependent manner. While no differences in the neurovascular barrier were observed until 3d, the barrier showed significant loss at 4d and 5d. However, the gliovascular barrier was significantly higher than the brain endothelial and neurovascular barriers until 2d. At 3d no significant difference was observed between any groups. However, gliovascular barrier was significantly lower than brain endothelial barrier at 4d and 5d. At 2d brain endothelial barrier was 73.4 ± 1.3%, neurovascular barrier was 73.43 ± 0.5% and gliovascular barrier was 91.99 ± 2.5%. At 4d brain endothelial barrier was 78.74 ± 2.3%, neurovascular barrier was 62.22 ± 3.8% and gliovascular barrier was 38.58 ± 1.6% (Figure 1E, Additional file 1: Figure S1E).

While the rank order of this experiment at 2d was HBMEC3=HBMEC3/SHSY-5Y<HBMEC3/HFA, at 4d the rank order was HBMEC3>HBMEC3/SHSY-5Y>HBMEC3/HFA.

##### Hypoxia

A significant decrease in hypoxia challenged neurovascular barrier compared to brain endothelial barrier was observed from 2d until 5d. However, hypoxia challenged gliovascular barrier was significantly lower compared to brain endothelial barrier from 1d until 5d. The differences between hypoxia challenged brain endothelial, neurovascular and gliovascular barriers were apparent at 3d and 4d. Hypoxia challenged neurovascular and gliovascular barriers were significantly lower compared to brain endothelial barrier at 3d and reached maximal at 4d. At 4d brain endothelial barrier was 63.71 ± 0.9%, neurovascular barrier was 36.13 ± 1.3% and gliovascular barrier was 11.4 ± 0.2% (Figure 1F, Additional file 1: Figure S1F).

The rank order of this experiment was HBMEC3>HBMEC3/SHSY-5Y>HBMEC3/HFA.

##### OGDR

Similar to aglycemia, OGDR challenged brain endothelial, neurovascular and gliovascular barriers showed differential responses in a time dependent manner. OGDR challenged neurovascular barrier was significantly higher than HBMEC3 until 3d, no differences were observed at 4d and 5d. While the barrier of OGDR challenged gliovascular barrier was significantly higher than brain endothelial barrier until 3d, at 4d the barrier was significantly lower than brain endothelial barrier. At 2d the barrier of brain endothelial cells was 74.08 ± 1.1%, neurovascular barrier was 99.03 ± 1.5% and gliovascular barrier was 180 ± 6.2%. At 4d brain endothelial barrier was 85.83 ± 1.6%, neurovascular barrier was 78.89 ± 5% and gliovascular barrier was 44.05 ± 5.7% (Figure 1G, Additional file 1: Figure S1G).

The rank order at 2d was HBMEC3<HBMEC3/SHSY-5Y<HBMEC3/HFA, and the rank order at 4d was HBMEC3=HBMEC3/SHSY-5Y>HBMEC3/HFA.

### Neuronal and astrocytic interactions modulate brain endothelial angiogenic property during in vitro ischemic metabolic stresses

#### Effect of metabolic stresses on brain endothelial angiogenic potential

**Figure 2:**
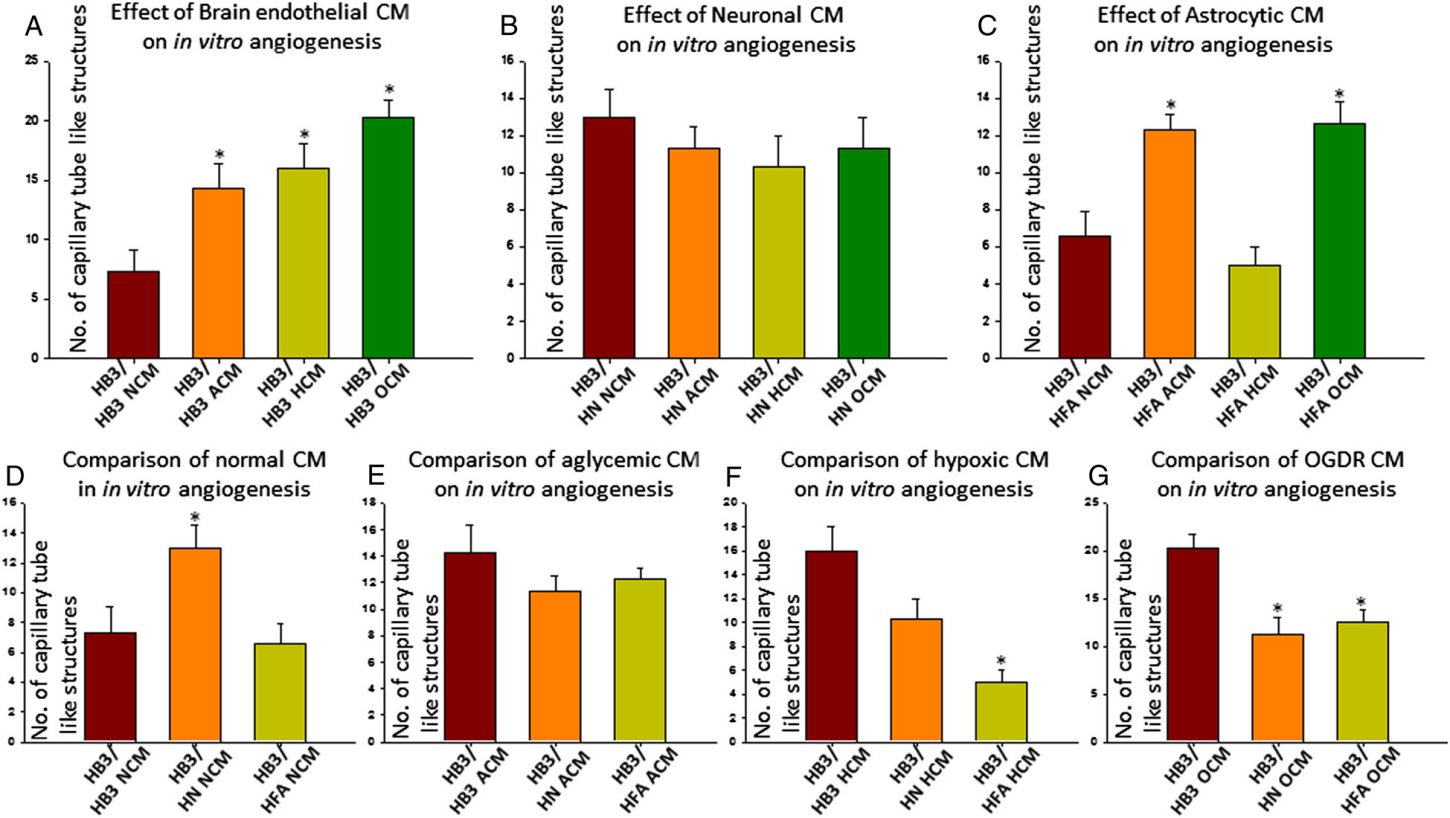
**Differential effects of metabolic stresses on neuronal and astrocytic interactions in modulating brain endothelial angiogenic potential. A**. *Effect of metabolically stressed brain endothelial CM (self-conditioned medium) on brain endothelial angiogenesis*. Significant increase in number of brain endothelial vessel like structures were observed with self-CM obtained from aglycemic, hypoxic and OGDR treated brain endothelial cells compared to untreated brain endothelial CM. **B**. *Effect of metabolically stressed neuronal CM on brain endothelial angiogenesis*. No significant difference in number of vessel like structures were observed with neuronal aglycemic, hypoxic or OGDR CM compared to untreated neuronal CM. **C**. *Effect of metabolically stressed astrocytic CM on brain endothelial angiogenesis*. Significant increase in number of vessel like structures were observed with astrocytic aglycemic and OGDR CM compared to untreated astrocyte CM. No significant differences were observed between astrocytic hypoxic CM and untreated astrocytic CM. **D**. *Comparison of untreated CM*. A significant increase in vessel like structures were observed with untreated neuronal CM compared to untreated brain endothelial CM. No differences between untreated astrocytic CM and untreated brain endothelial CM was observed. **E**. *Comparison of aglycemic CM*. No significant differences were observed between aglycemic brain endothelial, neuronal and astrocytic CM. **F**. *Comparison of hypoxic CM*. A significant decrease in vessel like structures were observed with hypoxic neuronal and astrocytic CM compared to hypoxic brain endothelial CM. **G**. *Comparison of OGDR CM from brain endothelial cells, neurons and astrocytes*. A significant decrease in vessel like structures were observed with OGDR neuronal and astrocytic CM compared to OGDR brain endothelial CM. One way ANOVA with Bonferroni post testing was used to check significance between 2 specific groups. Un-paired t-test was used to check statistical significance between two groups.*P<0.05 is considered significantly different from controls.

Conditioned medium from aglycemic, hypoxic and OGDR challenged brain endothelial cells (self conditioned medium from metabolically stressed brain endothelial cells) significantly induced *in vitro* brain endothelial capillary tube formation on matrigel compared to normal conditioned medium from brain endothelial cells (Figure 2A).

#### Effect of metabolically stressed neurons on brain endothelial angiogenic potential

Brain endothelial cells treated with conditioned medium from metabolically stressed neurons did not show any significant differences between each other (Figure 2B).

#### Effect of metabolically stressed astrocytes on brain endothelial angiogenic potential

Interestingly, conditioned medium from metabolically challenged astrocytes showed differential angiogenic potential in brain endothelial cells. Conditioned medium from aglycemia and OGDR challenged astrocytes induced a significant increase in brain endothelial *in vitro* capillary tube like formation (Figure 2C).

#### Comparison between neuronal and astrocyte CM on brain endothelial angiogenic potential

##### Normal

While normal astrocytic CM did not affect brain endothelial angiogenic potential, neuronal CM significantly increased brain endothelial *in vitro* capillary tube formation compared to normal brain endothelial CM (Figure 2D).

##### Aglycemia

Neither aglycemia challenged neuronal CM nor astrocytic CM affected brain endothelial angiogenic potential *in vitro* compared to aglycemia challenged brain endothelial CM (Figure 2E).

##### Hypoxia

While brain endothelial cells treated with hypoxia challenged neuronal CM decreased *in vitro* capillary tube formation, it did not achieve statistical significance. However, brain endothelial cells treated with hypoxia challenged astrocytic CM significantly decreased the brain endothelial angiogenic potential compared to hypoxia challenged brain endothelial CM (Figure 2F).

##### OGDR

Both OGDR challenged neuronal and astrocytic CM significantly decreased brain endothelial *in vitro* capillary tube formation compared to OGDR challenged brain endothelial CM (Figure 2G).

### Brain endothelial, neurovascular and gliovascular adhesive interactions with lymphocytes are modulated by in vitro ischemic metabolic stresses

#### Effect of ischemic metabolic stresses on brain endothelial-lymphocyte adhesion

**Figure 3:**
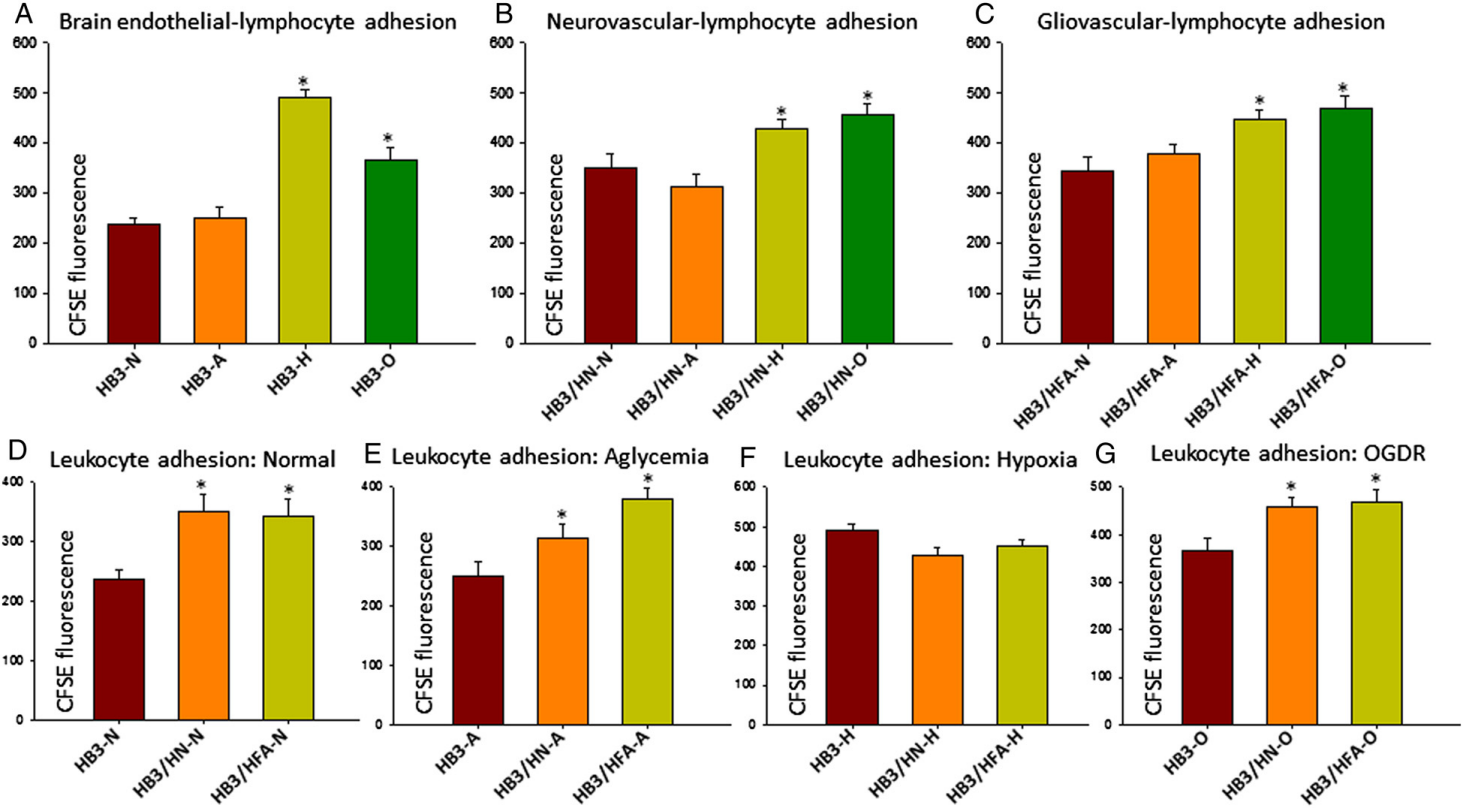
**Differential effects of metabolic stresses on brain endothelial, neurovascular and gliovascular-leukocyte adhesive interactions. A**. *Effect of metabolic stresses on brain endothelial-lymphocyte adhesion*. Hypoxic and OGDR treated brain endothelial cells significantly promoted lymphocyte adhesion compared to untreated and aglycemic brain endothelial cells. **B**. *Effect of metabolic stresses on neurovascular-lymphocyte adhesion*. Similar to brain endothelial-lymphocyte adhesion, hypoxia and OGDR significantly induced neurovascular-lymphocyte adhesion compared to untreated and aglycemic neurovascular-lymphocyte adhesion. **C**. *Effect of metabolic stresses on gliovascular-lymphocyte adhesion*. Similar to brain endothelial and neurovascular-lymphocyte adhesion, hypoxia and OGDR significantly induced gliovascular-lymphocyte adhesion compared to untreated and aglycemic gliovascular-lymphocyte adhesion. **D**. *Comparison of untreated brain endothelial, neurovascular and gliovascular-lymphocyte adhesion*. A significant increase in neurovascular and gliovascular-lymphocyte adhesion was observed compared to untreated brain endothelial cells. **E**. *Comparison of aglycemic brain endothelial, neurovascular and gliovascular-lymphocyte adhesion*. A significant increase in aglycemic neurovascular and gliovascular-lymphocyte adhesion was observed compared to aglycemic brain endothelial cells. **F**. *Comparison of hypoxic brain endothelial, neurovascular and gliovascular-lymphocyte adhesion*. No significant difference in hypoxic neurovascular and gliovascular-lymphocyte adhesion compared to hypoxic brain endothelial cells was observed. **G**. *Comparison of OGDR brain endothelial, neurovascular and gliovascular-lymphocyte adhesion*. A significant increase in OGDR neurovascular and gliovascular-lymphocyte adhesion was observed compared to OGDR brain endothelial cells. Un-paired t-test is used to check statistical significance between two groups. *P < 0.05 considered significantly different from controls.

While aglycemia did not affect brain endothelial-lymphocyte adhesive interactions, hypoxia and OGDR challenged brain endothelial cells significantly promoted lymphocyte adhesion *in vitro* compared to control (nomal) (Figure 3A).

#### Effect of ischemic metabolic stresses on neurovascular-lymphocyte adhesion

While aglycemia and hypoxia challenged neurovasculature did not affect lymphocyte adhesive interactions, OGDR challenged neurovasculature significantly promoted lymphocyte adhesion *in vitro* compared to control (normal) (Figure 3B).

#### Effect of ischemic metabolic stresses on gliovascular-lymphocyte adhesion

While aglycemia challenged gliovasculature did not affect lymphocyte adhesive interactions, both hypoxia and OGDR significantly increased lymphocyte adhesion *in vitro* compared to control (normal) (Figure 3C).

#### Comparison between brain endothelial, neurovascular and gliovascular-lymphocyte adhesive interactions

##### Normal

Interestingly, both neuronal and astrocytic interactions during normal conditions significantly promoted lymphocyte interactions compared with normal brain endothelial cells (Figure 3D).

##### Aglycemia

While aglycemia challenged neurovasculature did not significantly induce lymphocyte adhesion, aglycemia challenged gliovasculature significantly increased lymphocyte adhesion compared to aglycemia challenged brain endothelial cells (Figure 3E).

##### Hypoxia

While hypoxia challenged neurovasculature significantly decreased lymphocyte adhesion, hypoxia challenged gliovasculature did not affect lymphocyte adhesion compared to hypoxia challenged brain endothelial cells (Figure 3F).

##### OGDR

Both OGDR challenged neurovasculature and gliovasculature significantly promoted lymphocyte adhesion compared to OGDR challenged brain endothelial cells *in vitro* (Figure 3G).

#### Brain endothelial ECAM expression is modulated by metabolically stressed neurons and astrocytes during in vitro ischemic metabolic stresses

##### ICAM-1

**Figure 4:**
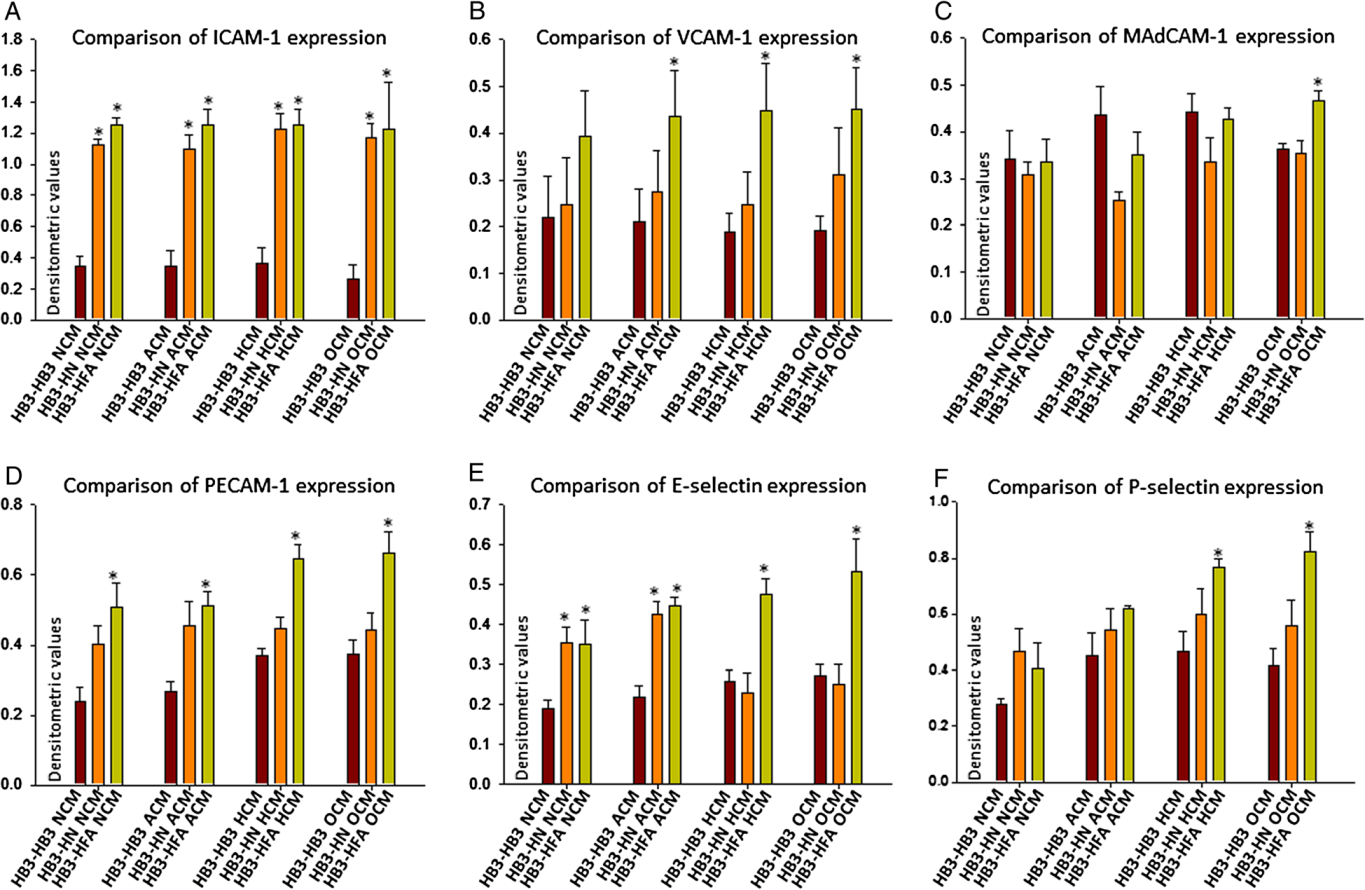
**Differential brain ECAM expression by metabolically stressed brain endothelial, neuronal and astrocyte secreted factors. A**. *Comparison of brain endothelial ICAM-1 expression*. A significant increase in brain endothelial ICAM-1 expression was observed with CM obtained from astrocytes and neurons from all conditions compared to respective brain endothelial CM. **B**. *Comparison of brain endothelial VCAM-1 expression*. While no significant increase in brain endothelial VCAM-1 expression was observed with any of neuronal CM, hypoxic and OGDR astrocytic CM significantly increased brain endothelial VCAM-1 expression compared to respective brain endothelial CM. **C**. *Comparison of brain endothelial MAdCAM-1 expression*. No significant difference in brain endothelial MAdCAM-1 expression was observed with CM obtained from astrocytes and neurons from any of conditions compared to respective brain endothelial CM. **D**. *Comparison of brain endothelial PECAM-1 expression*. While no significant difference in brain endothelial MAdCAM-1 expression was observed with any of neuronal CM, astrocytic CM from all conditions significantly increased brain endothelial PECAM-1 expression compared to respective brain endothelial CM. **E**. *Comparison of brain endothelial E-selectin expression*. A significant increase in brain endothelial E-selectin was observed with both normal and aglycemic neuronal and astrocytic CM compared to respective brain endothelial CM. However, while neither hypoxic nor OGDR neuronal CM increased brain E-selectin expression, both hypoxic and OGDR astrocytic CM significantly increased E-selectin expression compared to respective brain endothelial CM. **F**. *Comparison of brain endothelial P-selectin expression*. Except OGDR astrocytic CM, none of the other conditions significantly induced brain endothelial P-selectin expression compared to respective brain endothelial CM. Un-paired t-test was used to check significance between 2 specific groups. *P < 0.05 considered significantly different from controls.

CM from normal, aglycemia, hypoxia and OGDR challenged neurons and astrocytes significantly increased ICAM-1 expression on brain endothelial cells (Figure 4A, Additional file 2: Figure S2A, Additional file 3: Figure S3A).

##### VCAM-1

Neuronal or astrocytic normal CM did not induce VCAM-1 expression compared to normal brain endothelial CM. However, aglycemia, hypoxia and OGDR challenged astrocytic CM significantly induced VCAM-1 expression compared to respective CM from brain endothelial cells. No differences with CM from neurons in any of these conditions significantly affected VCAM-1 expression compared to respective CM from brain endothelial cells (Figure 4B, Additional file 2: Figure S2B, Additional file 3: Figure S3A).

##### MAdCAM-1

None of the treatment conditions except CM from aglycemia challenged neurons affected MAdCAM-1 expression in brain endothelia cells. CM from aglycemia challenged neurons significantly decreased MAdCAM-1 expression compared to aglycemia challenged brain endothelial CM (Figure 4C, Additional file 2: Figure S2C, Additional file 3: Figure S3B).

##### PECAM-1

CM from astrocytes from all treatment conditions significantly induced PECAM-1 expression compared to respective CM from brain endothelial cells. CM for neurons did not show any significant differences in PECAM-1 expression (Figure 4D, Additional file 2: Figure S2D, Additional file 3: Figure S3B).

##### E-Selectin

No significant difference in E-selectin expression was observed between CM from normal neurons or astrocytes compared to CM from normal brain endothelial cells. However, both aglycemia challenged neurons and astrocytes significantly induced E-selectin expression compared to aglycemia challenged brain endothelial cells. Interestingly, while hypoxia or OGDR challenged neurons did not show any difference in MAdCAM-1 expression, hypoxia and OGDR challenged astrocytes significantly induced MAdCAM-1 expression compared to respective CM from brain endothelial cells (Figure 4E, Additional file 2: Figure S2E, Additional file 3: Figure S3A).

##### P-Selectin

While CM media from normal astrocytes significantly induced P-selectin expression, CM from normal neurons did not induce significant P-selectin expression (p = 0.09). Neither aglycemia challenged CM from neurons nor astrocytes affected P-selectin expression compared to respective CM from brain endothelial cells. Furthermore, neither hypoxia nor OGDR challenged CM from neurons induced P-selectin expression compared to respective CM from brain endothelial cells. However, both hypoxia and OGDR challenged astrocytic CM induced P-selectin expression compared to respective CM from brain endothelial cells (Figure 4F, Additional file 2: Figure S2F, Additional file 3: Figure S3B).

## Discussion and conclusions

Pathology of cerebral ischemia involves a highly complex association of inflammatory and immune events at the neurovascular and gliovascular units [14,20,22,23,31,34,35]. The BBB, the crucial entity that shields neurovascular and gliovascular units from the systemic challenges, is highly compromised during ischemic insult [36-39]. Ischemic insult initiates an extensive chain of events at these units both at biophysical and biochemical levels [7,8,38-43]. The signaling events and interactions between the cell types in these units during cerebral ischemia are extremely difficult to elucidate. Hence, the 3D models of neurovascular and gliovascular interactions provide a highly useful system to understand endothelial responses in modulating the BBB permeability that affects cerebral edema, post ischemic brain angiogenesis (associated with the survival of stroke patients) and leukocyte interactions that aggravate ischemia reperfused (I/R) stroke brain damage [28,35,36,44].

While several previous reports have shown that inflammation and hypoxia leads to BBB breakdown [45], it was not still clear whether glucose unavailability or oxygen unavailability or a combination of these conditions are implicated in this process. In our brain endothelial, gliovascular and neurovascular barrier studies, a very interesting barrier regulation by neurons and astrocytes during the metabolic stresses of cerebral ischemia was observed. While normal brain endothelial responses to the metabolic stresses were only modest, gliovascular and neurovascular barrier showed a dramatic increase in barrier under OGDR conditions. Interestingly, while hypoxic neurovascular and gliovascular barrier showed either no effect or decreased barrier respectively, aglycemic neurovascular and gliovascular barriers showed modest increase in barrier as compared to OGDR. These results clearly indicate that aglycemia might be the underlying component in increased barrier suggesting that OGDR can produce a highly adaptive and protective barrier phenotype. These results indicate that during ischemic stress, neuronal and astrocytic responses might synergize with OGDR to attenuate BBB permeability and in the recovery of the barrier. However, these stresses coupled with unrestricted levels of cytokines, chemokines and proteases in the ischemic infarct might skew the adaptive nature of BBB and lead to increased permeability [46,47].

One of the main reasons in OGDR mediated barrier increase can be explained in terms energy conservation. During aglycemic cell stress, lack of glucose stimulates endothelium to conserve their energy sources by shutting down the cellular metabolic process and diverting the cellular energy pools to maintain barrier integrity and in endothelial reparative processes. This process is observed during the cellular attempt to maintain an adaptive phenotype to sustain throughout the cell stress as long as possible and to prepare for an ordered apoptotic cell death rather than necrosis [48]. Similar to our previous observations, we expect that during aglycemic conditions, the endothelium attempts to protect and maintain barrier integrity as a primary event in the attenuating brain edema during cerebral ischemia [24]. While aglycemia results in this protective phenotype, availability of glucose in hypoxic stress might lead to signaling events in an opposite direction. These results indicate that availability of either oxygen or glucose alone might play a pivotal role in determining endothelial phenotype during cerebral ischemia. However, a combination of these mutual opposite barrier modulating events resulted in a more beneficial barrier phenotype due to the underlying signaling events initiated by aglycemic stress. The molecular signaling events involved in OGDR mediated improved barrier needs to be further investigated. These responses were also observed in the *in vitro* angiogenesis assay during these metabolic stresses.

In our *in vitro* angiogenesis experiments, a significant impact of metabolically stressed astrocytic and neuronal secreted factors on brain endothelial angiogenic potential was observed. While aglycemia, hypoxia and OGDR significantly increased brain endothelial angiogenesis, metabolic stressed neuronal CM did not show any effect compared to control (normal neuronal CM). However, aglycemic and OGDR astrocytic CM significantly increased brain endothelial angiogenesis. A comparison between different groups with the same treatment showed that, while normal neuronal CM significantly increased capillary tube formation, normal astrocyte CM did not compared to normal brain endothelial CM. Interestingly, both hypoxic and OGDR CM from astrocytes and neurons significantly decreased capillary tube formation. Since increased angiogenesis is highly required for the reparative process, these results were puzzling. However, we expect that the factors secreted by metabolically stressed neurons and astrocytes might be responsible for this effect. It is also clear that while aglycemia plays a major role in modulating OGDR astrocytic and neuronal barrier, hypoxia plays a prominent role in modulating brain endothelial angiogenic potential by OGDR astrocytes and neurons. The pathophysiological role of neuronal and astrocytic physical interactions and the secreted cell signaling mediators in mediating brain endothelial angiogenic potential needs to be investigated.

Post ischemic brain angiogenesis has been strongly associated with stroke mortality and outcomes. While increased post I/R brain angiogenesis is associated with better outcome, decreased angiogenesis is associated with increased mortality [28,49-51]. Neurovascular and gliovascular interactions display an extremely complex internal and external cross talk in CNS vascularization. Recent reports have shown that both astrocytic and neuronal tropic factors can not only modulate endothelial pro-angiogenic activity directly but also by recruiting hematopoietic precursors from the circulation [52,53]. However, the inherent mechanisms that control the ischemic brain angiogenesis at the neurovascular units and the cellular mechanisms and associations that are required for improved cerebral blood flow are not yet known. One of the most important aspects in the angiogenesis at the bioenergetics level is the conservation of energy [54,55]. For example, during less severe ischemic brain damage, angiogenesis can help to attempt increase blood supply and results in the association with increased survival. However, if the severity is too large, induction of angiogenesis will only result in the depletion of energy stores and in the aggravation of brain damage. This is similar to the function of poly ADP ribose polymerase-1 (PARP-1) which at sub injury level helps in the repair of damaged DNA and the recovery of the cell. However, during extensive cellular damage, PARP-1 utilizes the energy reserves and results in necrotic cell death and increased tissue injury [56]. The role of neurons and astrocytes at the neurovascular and gliovascular units in governing post-stroke brain angiogenesis and how their intimate association modulates immune responses during cerebral ischemia are not yet clear [57,58].

During cerebral ischemia, increased reactive oxygen species, cytokines and microglial responses leads to a highly inflamed endothelium which actively participates in systemic leukocytes adhesion and infiltration into the brain [59,60]. In the extensively damaged BBB of the necrotic infarct core, leukocyte infiltration is ECAM independent [61,62]. However, in the relatively intact BBB of the infarct penumbra, leukocyte infiltration proceeds via ECAM dependent [61,62]. Increased expression of ECAMs is one of the typical features of reactive brain endothelium [62]. We have previously observed that metabolic stresses of cerebral ischemia contribute differentially to the expression profiles of brain ECAMs [25]. However, it is not clear how metabolically stressed neurons and astrocytes contribute to this process. While astrocytes are inert to immune cell interactions, reactive astrocyte secrete several proteases and cytokines that modulate endothelial properties in driving leukocyte interactions [44,63,64]. Furthermore, neurons were also shown to support leukocyte adhesion and mediated damage [44,46,63,65-69]. Hence, it becomes apparent that several intercellular signaling mechanisms might operate in the neurovascular and gliovascular units that can potentially modulate brain endothelial responses to leukocyte recruitment during cerebral ischemia.

In our brain endothelial, gliovascular and neurovascular lymphocyte adhesion assay, while hypoxic and OGDR brain endothelium actively supports lymphocyte adhesion, astrocyte CM or neuronal CM during any metabolic stress did not affect brain endothelial lymphocyte adhesion compared to normal CM. However, we observed from a comparison between the groups with the same treatment that both neurovascular and gliovascular interactions show significant increase in the lymphocyte adhesion correlating with increased brain endothelial ICAM-1 expression with normal astrocytic or neuronal CM. Interestingly, while aglycemia and hypoxic neuronal or astrocytic CM did not show any significant difference from aglycemic or hypoxic brain endothelium in lymphocyte adhesion, OGDR CM from both astrocytes and neurons significantly increased lymphocyte adhesion compared to OGDR brain endothelium, clearly indicating that cerebral ischemia induces neurovascular and gliovascular adhesive interactions with lymphocytes.

One of the most interesting findings in our current study was observed in ICAM-1 expression. A significant increase in the basal expression of ICAM-1 was observed when treated with astrocyte or neuronal CM as compared to brain endothelial CM even under normal conditions. This indicates that ICAM-1 might be a principal player in mediating physiological neurovascular or gliovascular immune cell interactions. Since ICAM-1 was also used as an inflammatory endothelial marker [70], the expression of ICAM-1 should exceed the neurovascular or gliovascular ICAM-1 basal expression levels to participate in pathophysiological leukocyte endothelial interactions. While our data suggests that ICAM-1 might be key player in leukocyte-neurovascular and gliovascular adhesive interactions under physiological conditions, VCAM-1 and PECAM-1 which showed significant increases during OGDR astrocyte CM might play a potential role during pathophysiological conditions. Furthermore, E-selectin which showed a prominent increase with OGDR astrocyte CM might play an important role in initiating neutrophil interactions to brain endothelium [71]. While several treatment combinations may be required to further understand the roles of these ECAM modulation by neurovascular and gliovascular units in initiation and propagation of leukocyte interactions, our current study provides a basic understanding on how metabolic stresses can affect neuronal and astrocytic interactions in modulating brain endothelial interactions with lymphocytes.

## Materials and methods

### Cell culture

HBMEC3, a human brain endothelial cell line (provided by Dr. Anat Erdreich-Epstein, Univ. of Southern California) was cultured in Roswell Park Memorial Institute (RPMI) medium supplemented with 10% fetal calf serum and 1% penicillin/streptomycin/Amphotericin. HFA, a human astrocyte cell line (provided by Dr. Danica Stanimirovic, Univ. Ottawa), and SHSY-5Y, (HN) a human neuronal cell line, were cultured in Dulbecco Modified Eagles medium (DMEM) supplemented with 10% FCS and 1% PSA [24,25].

### Neurovascular and Gliovascular culture conditions

Neurovascular barrier was maintained by culturing HBMEC3 in the upper chamber (insert) with 10% RPMI and SHSY-5Y in the lower chamber with 10% DMEM. Similarly, gliovascular barrier was maintained by culturing HBMEC3 in the upper chamber (insert) with 10% RPMI and HFA in the lower chamber with 10% DMEM.

### Neurovascular Barrier treatment conditions

HBMEC3 was cultured on the inside of the 8.0 μm insert and SHSY-5Y on the other side (bottom side) of the insert. This allows the formation of contact dependent neurovascular barrier. Six hours after *in vitro* N, A, H or O simulation, inserts with neurovascular barrier were incubated in 10% RPMI in the insert and 10% DMEM in the lower chamber. The barrier was measured every 24 h. The time point of addition of media after simulation was considered 0 h and also the baseline for all other time points in the group.

### Gliovascular Barrier treatment conditions

HBMEC3 was cultured on the inside of the 8.0 μm insert and HFA was cultured on the other side (bottom side) of the insert. This allows the formation of contact dependent gliovascular barrier. Six hours after *in vitro* N, A, H or O simulation, inserts with gliovascular barrier were incubated in 10% RPMI in the insert and 10% DMEM in the lower chamber. The barrier was measured every 24 h. The time point of addition of media after simulation was considered 0 h and also the baseline for all other time points in the group [24].

### Brain endothelial barrier treatment conditions

After challenging with normal, aglycemia, hypoxia or OGD for 6 h, cells were incubated in 10% RPMI in the insert and 10% DMEM in the lower chamber under normoxic conditions to match gliovascular and neurovascular treatment conditions as described above. This treatment condition allowed to compensate for media differences among brain endothelial, gliovascular and neurovascular barrier and to compare the barrier readings among brain endothelial, gliovascular and neurovascular barriers. The barrier was recorded with an EVOM meter every 24 h as previously described [24]. The time point of addition of media after simulation was considered 0 h and also the baseline for all other time points in the group.

### Human lymphocyte isolation

Lymphocytes were isolated from peripheral blood as previously described by ficoll-hypaque (1.077) method. Lymphocytes were activated using PMA/calcium ionophore as previously described [46].

### Carboxyfluorescein succinimidyl ester (CFSE) labeling

Lymphocytes were suspended in PBS with 5 mM CFSE for 10 min with occasional mixing. After 10 min cells were kept on ice and suspended in ice cold PBS. Later, cells were washed 3 times and suspended in 10% RPMI and incubated in the incubator under normal culture conditions.

### Brain endothelial-Lymphocyte adhesion

In order restrict any lymphocyte trans-endothelial migration across brain endothelial, gliovascular or neurovascular barriers (cultured on 8.0 μm pore inserts), we used 1.0 μm pore inserts in the lymphocyte adhesion assays. We expected that a pore size restriction (8.0 μm vs. 1.0 μm) might help restrict migration of lymphocytes across these barriers and can produce a more accurate measurement of lymphocyte adhesion to the brain endothelial, gliovascular and neurovascular barriers.

HBMEC3 was cultured inside of the 1.0 μM insert and was subjected to N, A, H or O. After treatment, cells were incubated in normal medium for 24 h. Later, CFSE labeled, PMA/calcium ionophore activated (for 24 h) human lymphocytes were added to each insert and incubated for 1 h in the incubator. Later, inserts were washed 3 times with PBS and fluorescence of attached lymphocytes was measured by a fluorescent plate reader with fixed gain. To decrease deviation/error in these experiments adhesion assays were performed at the same time. Gain remained fixed in all adhesion assays.

### Neurovascular-Lymphocyte adhesion

HBMEC3 was cultured on the inside of the 1.0 μm insert and SHSY-5Y was cultured on the other side (bottom side) of the insert. After *in vitro* N, A, H or O for 6 h inserts with neurovascular barrier were incubated in 10% RPMI in the insert and 10% DMEM in the lower chamber. Later, CFSE labeled, PMA/calcium ionophore activated (for 24 h) human lymphocytes were added to each insert and incubated for 1 h in the incubator. Later, inserts were washed 3 times with PBS and fluorescence of attached lymphocytes was measured by a fluorescent plate reader with fixed gain.

### Gliovascular-Lymphocyte adhesion

HBMEC3 was cultured on the inside of the 8.0 μm insert and HFA was cultured on the other side (bottom side) of the insert. This allows the formation of contact dependent gliovascular barrier. After *in vitro* N, A, H or O for 6 h inserts with gliovascular barrier were incubated in 10% RPMI in the insert and 10% DMEM in the lower chamber. Later, CFSE labeled, PMA/calcium ionophore activated (for 24 h) human lymphocytes were added to each insert and incubated for 1 h in incubator. Later, inserts were washed 3 times with PBS and fluorescence of attached lymphocytes was measured by a fluorescent plate reader with fixed gain.

### Conditioned media

HBMEC3, SHSY-5Y and HFA were subjected to N, A, H or O for 6 h followed by incubating in normal culture conditions. After 24 h, N, A, H or O conditioned media from HBMEC3, SHSY-5Y or HFA were collected and filtered through a 0.22 μm filter and stored at −80 °C until use.

### Western blot analysis

HBMEC3 cells were treated with conditioned media obtained from HBMEC3 (N, A, H or O), HFA (N, A, H or O) or SHSY-5Y (N, A, H or O) for 24 h. Later, cells were collected in a non-reducing sample buffer with a protease inhibitor cocktail. Cells were lysed using a sonicator and were resolved in an SDS-PAGE and immunoblotted with ICAM-1, VCAM-1, MAdCAM-1, PECAM-1, E-Selectin and P-Selectin.

### Antibodies

ICAM-1 (Cat. No. 09351D, 1:500) was purchased from Pharmingen, San Diego, CA, USA; VCAM-1 (Cat. No. 553329, 1:500) was purchased from BD Biosciences, San Diego, CA, USA; MAdCAM-1 (MECA-367, Cat. No. 09721D, 1:500) was purchased from BD Pharmingen, San Diego, CA, USA; PECAM-1 (Cat. No. 550274, 1:500) was purchased from BD Pharmingen; E-selectin (Cat. No. 553748, 1:500) was purchased from BD Biosciences; P-selectin (Cat. No. 09480D, 1:1000) was purchased from Pharmingen.

### In vitro angiogenesis

HBMEC3 cells were treated with conditioned media obtained from HBMEC3 (N, A, H or O), HFA (N, A, H or O) or SHSY-5Y (N, A, H or O) for 24 h. Later, cells were trypsinized and plated in matrigel for 3 h on 96-well black walled plates. Capillary tube like structure formation on the matrigel was photographed at the concave center of the well and the number of vessel like structures in each image was counted [72].

### Statistics

Graphpad-3 InStat™ software was used to perform statistical analyses. Repeated measures ANOVA was used for barrier studies. One way ANOVA or repeated measures ANOVA each with Dunnett's' post-hoc test or Bonferroni post-test were used to determine statistical significance. Sigmaplot™ was used to generate plots. *p < 0.05 was considered to be statistically significant, **p < 0.01 very significant, and ***p < 0.001 highly significant.

## Abbreviations

ICAM-1: Intercellular adhesion molecule-1; VCAM-1: Vascular cell adhesion molecule-1; PECAM-1: Platelet endothelial cell adhesion molecule-1; MAdCAM-1: Mucosal addressin cell adhesion molecule-1; ECAM: Endothelial cell adhesion molecule; PARP-1: Poly-ADP ribose polymerase-1; N: Normoxia normoglycemia; H: Hypoxia; A: Aglycemia; O (OGDR): Oxygen glucose deprivation and reoxygenation; EVOM: Epithelial Volt-ohm meter; CFSE: Carboxyfluorescein succinimidyl ester; CM: Conditioned medium.

## Competing interests

The authors declare that they have no competing interests.

## Authors’ contributions

GVC conceived the idea, planned and performed the experiments, analyzed and interpreted the data, wrote and edited the manuscript. JSA edited the manuscript and facilitated the research. All authors read and approved the final manuscript.

## Supplementary Material

Additional file 1: Figure S1 Differential effects of metabolic stresses on brain endothelial, neurovascular and gliovascular barriers. A. *Brain endothelial barrier*. HB3-N: Normal brain endothelial barrier, HB3-A: Aglycemic brain endothelial barrier, HB3-H: Hypoxic brain endothelial barrier, HB3-O: OGDR brain endothelial barrier. B. *Neurovascular barrier*. HB3/HN-N: Normal neurovascular barrier, HB3/HN-A: Aglycemic neurovascular barrier, HB3/HN-H: Hypoxic neurovascular barrier, HB3/HN-O: OGDR neurovascular barrier. C. *Gliovascular barrier*. HB3/HFA-N: Normal gliovascular barrier, HB3/HFA-A: Aglycemic gliovascular barrier, HB3/HFA-H: Hypoxic gliovascular barrier, HB3/HFA-O: OGDR gliovascular barrier. D. *Comparison of untreated brain endothelial, neurovascular and gliovascular barriers*. E. *Comparison of aglycemic brain endothelial, neurovascular and gliovascular barriers*. F. *Comparison of hypoxic brain endothelial, neurovascular and gliovascular barriers*. G. *Comparison of OGDR brain endothelial, neurovascular and gliovascular barriers*. Repeated measures ANOVA for repeated time course measurements from 0 h baseline. Values are expresses in percent ± SEM. Un-paired t-test was used to check significance between groups at the same time point. ^#^P < 0.05 is considered significantly different from controls at the same time point. Click here for file

Additional file 2: Figure S2 Differential brain ECAM expression by metabolically stressed brain endothelial, neuronal and astrocyte secreted factors. A. *ICAM-1.* No significant difference in brain endothelial ICAM-1 expression was observed by any of brain endothelial, astrocytic or neuronal CM compared to respective CM. B. *VCAM-1*. A significant increase in brain endothelial VCAM-1 expression was observed by hypoxic and OGDR treated brain endothelial CM (self-conditioned medium) compared to untreated brain endothelial CM. None of the neuronal or astrocytic CM induced brain endothelial VCAM-1 expression compared to respective untreated CM C. *MAdCAM-1*. None of the CM induced brain endothelial MAdCAM-1 expression compared to respective untreated CM. D. *PECAM-1*. None of the CM induced brain endothelial PECAM-1 expression compared to respective untreated CM. E. *E-selectin*. None of the CM induced significant brain endothelial E-selectin expression compared to respective untreated CM. F. *P-selectin*. While none of the CM from brain endothelial cells or neurons significantly induced P-selectin expression of brain endothelial cells, aglycemic, hypoxic and OGDR astrocytic CM significantly induced brain endothelial p-selectin expression compared to untreated astrocytic CM. Un-paired t-test was used to check significance between 2 specific groups. *P < 0.05 considered significantly different from controls. Click here for file

Additional file 3: Figure S3 Western blot images of brain ECAM expression by metabolically stressed neurons and astrocytes. Panels A and B were presented based on the sequence of cell lysates loading. A. ICAM-1, VCAM-1, P-Selectin and Actin expression. Order of protein loading sequence in *panel A: Lysates obtained from brain endothelial cells treated with metabolically stressed brain endothelial conditioned medium, conditioned medium from metabolically stressed neurons and conditioned medium from metabolically stressed astrocytes*. Expression of brain endothelial ICAM-1, VCAM-1 and P-Selectin was normalized to actin (loading control). B. MAdCAM-1, PECAM-1, E-Selectin and Actin expression. Order of protein loading sequence in *panel B: Lysates obtained from brain endothelial cells treated with metabolically stressed brain endothelial conditioned medium, conditioned medium from metabolically stressed astrocytes and conditioned medium from metabolically stressed neurons.* Expression of brain endothelial MAdCAM-1, PECAM-1 and E-Selectin was normalized to actin (loading control). Click here for file
