# Supplementary figures and images for: Neuronal and astrocytic interactions modulate brain endothelial properties during metabolic stresses of in vitro cerebral ischemia
Source: Cell Commun Signal. 2014 Jan 17;12:7. doi: 10.1186/1478-811X-12-7 (PMC3927849; doi:10.1186/1478-811X-12-7)

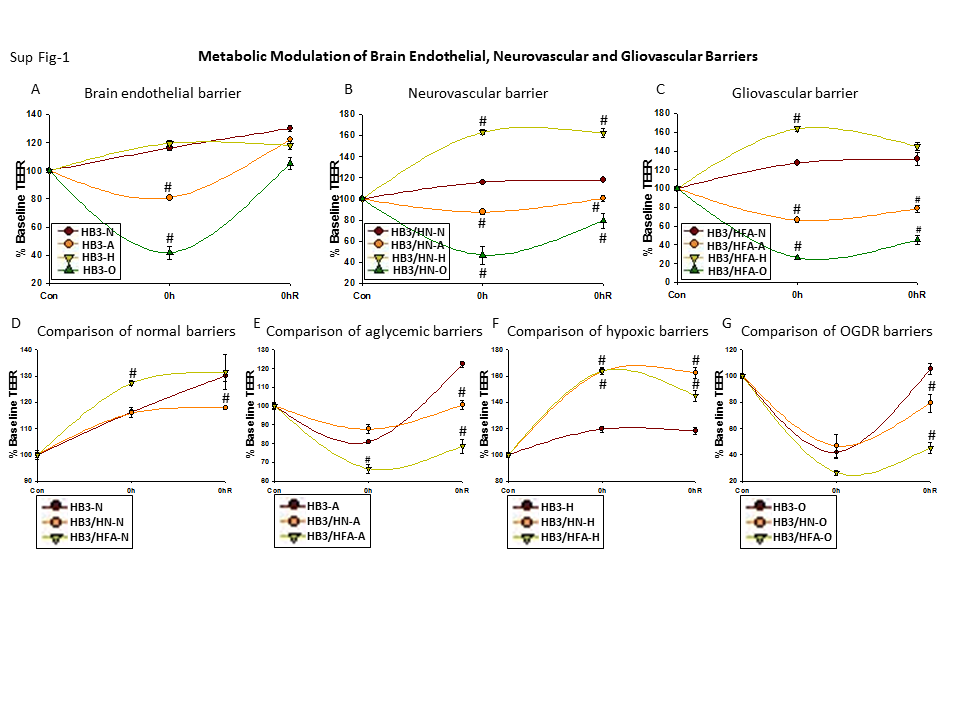

Supplement: Additional file 1: Figure S1 — Differential effects of metabolic stresses on brain endothelial, neurovascular and gliovascular barriers. A. Brain endothelial barrier. HB3-N: Normal brain endothelial barrier, HB3-A: Aglycemic brain endothelial barrier, HB3-H: Hypoxic brain endothelial barrier, HB3-O: OGDR brain endothelial barrier. B. Neurovascular barrier. HB3/HN-N: Normal neurovascular barrier, HB3/HN-A: Aglycemic neurovascular barrier, HB3/HN-H: Hypoxic neurovascular barrier, HB3/HN-O: OGDR neurovascular barrier. C. Gliovascular barrier. HB3/HFA-N: Normal gliovascular barrier, HB3/HFA-A: Aglycemic gliovascular barrier, HB3/HFA-H: Hypoxic gliovascular barrier, HB3/HFA-O: OGDR gliovascular barrier. D. Comparison of untreated brain endothelial, neurovascular and gliovascular barriers. E. Comparison of aglycemic brain endothelial, neurovascular and gliovascular barriers. F. Comparison of hypoxic brain endothelial, neurovascular and gliovascular barriers. G. Comparison of OGDR brain endothelial, neurovascular and gliovascular barriers. Repeated measures ANOVA for repeated time course measurements from 0 h baseline. Values are expresses in percent ± SEM. Un-paired t-test was used to check significance between groups at the same time point. #P < 0.05 is considered significantly different from controls at the same time point. [file 1478-811X-12-7-S1.tiff]

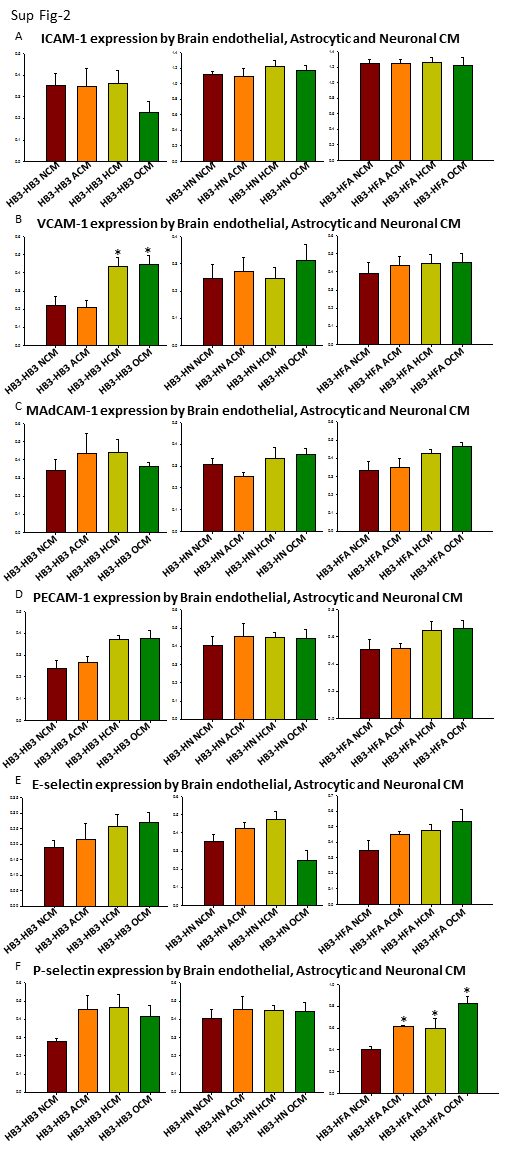

Supplement: Additional file 2: Figure S2 — Differential brain ECAM expression by metabolically stressed brain endothelial, neuronal and astrocyte secreted factors. A. ICAM-1. No significant difference in brain endothelial ICAM-1 expression was observed by any of brain endothelial, astrocytic or neuronal CM compared to respective CM. B. VCAM-1. A significant increase in brain endothelial VCAM-1 expression was observed by hypoxic and OGDR treated brain endothelial CM (self-conditioned medium) compared to untreated brain endothelial CM. None of the neuronal or astrocytic CM induced brain endothelial VCAM-1 expression compared to respective untreated CM C. MAdCAM-1. None of the CM induced brain endothelial MAdCAM-1 expression compared to respective untreated CM. D. PECAM-1. None of the CM induced brain endothelial PECAM-1 expression compared to respective untreated CM. E. E-selectin. None of the CM induced significant brain endothelial E-selectin expression compared to respective untreated CM. F. P-selectin. While none of the CM from brain endothelial cells or neurons significantly induced P-selectin expression of brain endothelial cells, aglycemic, hypoxic and OGDR astrocytic CM significantly induced brain endothelial p-selectin expression compared to untreated astrocytic CM. Un-paired t-test was used to check significance between 2 specific groups. *P < 0.05 considered significantly different from controls. [file 1478-811X-12-7-S2.tiff]

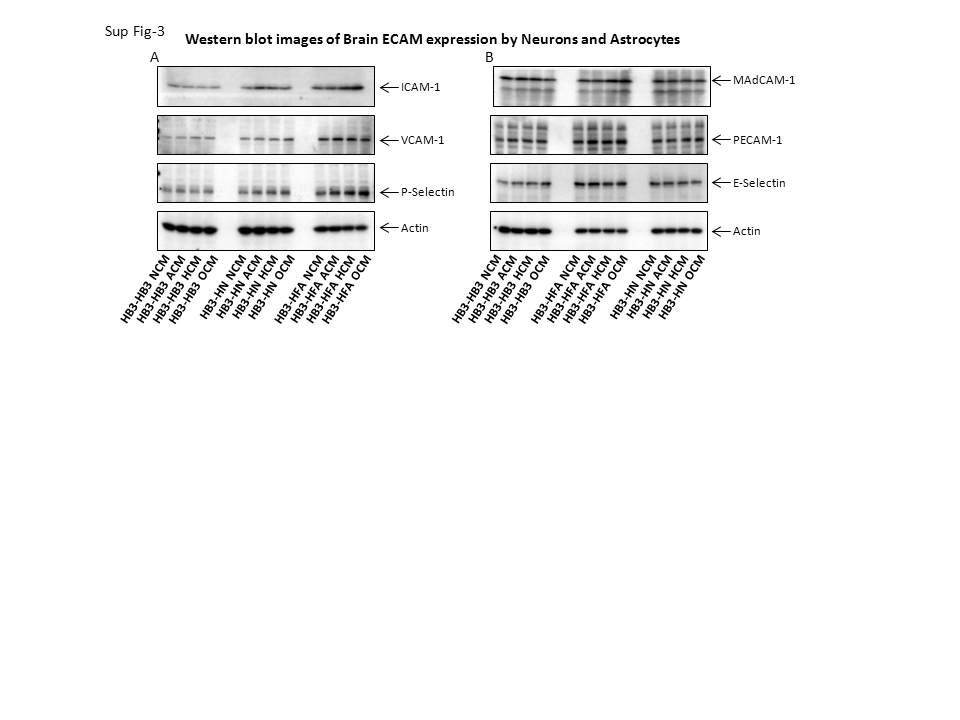

Supplement: Additional file 3: Figure S3 — Western blot images of brain ECAM expression by metabolically stressed neurons and astrocytes. Panels A and B were presented based on the sequence of cell lysates loading. A. ICAM-1, VCAM-1, P-Selectin and Actin expression. Order of protein loading sequence in panel A: Lysates obtained from brain endothelial cells treated with metabolically stressed brain endothelial conditioned medium, conditioned medium from metabolically stressed neurons and conditioned medium from metabolically stressed astrocytes. Expression of brain endothelial ICAM-1, VCAM-1 and P-Selectin was normalized to actin (loading control). B. MAdCAM-1, PECAM-1, E-Selectin and Actin expression. Order of protein loading sequence in panel B: Lysates obtained from brain endothelial cells treated with metabolically stressed brain endothelial conditioned medium, conditioned medium from metabolically stressed astrocytes and conditioned medium from metabolically stressed neurons. Expression of brain endothelial MAdCAM-1, PECAM-1 and E-Selectin was normalized to actin (loading control). [file 1478-811X-12-7-S3.tiff]
